# Analgesic and Antidepressant Effects of Oltipraz on Neuropathic Pain in Mice by Modulating Microglial Activation

**DOI:** 10.3390/jcm8060890

**Published:** 2019-06-21

**Authors:** Andrés Felipe Díaz, Sara Polo, Núria Gallardo, Sergi Leánez, Olga Pol

**Affiliations:** 1 Grup de Neurofarmacologia Molecular, Institut d’Investigació Biomèdica Sant Pau, Hospital de la Santa Creu i Sant Pau, 08025 Barcelona, Spain; andresfelipe.diaz@e-campus.uab.cat (A.F.D.); sara.polor@e-campus.uab.cat (S.P.); u1934609@campus.udg.edu (N.G.); sergi.leanez@uab.es (S.L.); 2 Institut de Neurociències, Universitat Autònoma de Barcelona, 08193 Barcelona, Spain

**Keywords:** analgesia, inflammation, microglia, neuropathic pain, oxidative stress, oltipraz

## Abstract

Nerve injury provokes microglial activation, contributing to the sensory and emotional disorders associated with neuropathic pain that do not completely resolve with treatment. In C57BL/6J mice with neuropathic pain induced by chronic constriction of the sciatic nerve (CCI), we evaluated the effects of oltipraz, an antioxidant and anticancer compound, on (1) allodynia and hyperalgesia, (2) microglial activation and pain signaling pathways, (3) oxidative stress, and (4) depressive-like behaviors. Twenty-eight days after surgery, we assessed the effects of oltipraz on the expression of CD11b/c (a microglial marker), phosphoinositide 3-kinase (PI3K)/ phosphorylated protein kinase B (p-Akt), nuclear factor-κB (NF-κB) transcription factor, and mitogen activated protein kinases (MAPK) in the spinal cord, hippocampus, and prefrontal cortex. Our results show that oltipraz alleviates neuropathic pain by inhibiting microglial activation and PI3K/p-Akt, phosphorylated inhibitor of κBα (p-IκBα), and MAPK overexpression, and by normalizing and/or enhancing the expression of antioxidant proteins, nuclear factor erythroid derived-2-related factor 2 (Nrf2), heme oxygenase 1 (HO-1), and NAD(P)H:quinone oxidoreductase-1 (NQO1) in the spinal cord. The inhibition of microglial activation and induction of the Nrf2/HO-1/NQO1 signaling pathway in the hippocampus and/or prefrontal cortex may explain the antidepressant effects of oltipraz during neuropathic pain. These data demonstrate the analgesic and antidepressant effects of oltipraz and reveal its protective and antioxidant properties during chronic pain.

## 1. Introduction

Chronic neuropathic pain caused by nerve injury provokes peripheral and central sensitization as well as several emotional disorders [1,2]. Central sensitization is mainly produced by activated microglia and the release of bioactive mediators, including phosphoinositide 3-kinase (PI3K)/ protein kinase B (Akt), nuclear factor-κB (NF-κB), and mitogen-activated protein kinases (MAPK), which facilitate pain signaling in the peripheral and central nervous system, thus contributing to neuropathic pain [3,4,5,6].

The implication of spinal microglia in the progression of neuropathic pain is well recognized [7,8], but the role of microglia in supraspinal structures after peripheral nerve injury is less understood. Recent studies have demonstrated that persistent neuropathic pain caused by sciatic nerve injury, in addition to activating spinal microglia, provokes microglial activation in several brain areas, such as the hippocampus, amygdala, poster lateral nucleus of the thalamus, and anterior cingulate cortex, contributing to the sensory and emotional disorders associated with chronic pain [9,10,11,12]. In contrast, previous studies performed seven days post nerve ligation only revealed microglial activation in the spinal cord but not in the hippocampus, amygdala, and periaqueductal gray, which may be related to one week being insufficient time to activate microglia in supraspinal regions [13]. The administration of microglial inhibitors, such as minocycline, inhibits neuropathic pain and depressive-like behaviors [14]. 

Phosphorylated extracellular signal regulated kinase 1/2 (p-ERK 1/2) and p38 MAPK levels increase in the spinal cord of nerve-injured mice [15,16], and the inhibition of ERK 1/2 and p38 MAPK expression diminishes mechanical and thermal hyperalgesia [4,7,17]. Microglial activation also results in the production of other bioactive intermediaries, such as the PI3K/p-Akt and NF-κB signaling pathways, which also contribute to neuropathic pain [5]. As a result, increased protein levels of PI3K/p-Akt and phosphorylated inhibitor of κBα (p-IκBα) have been demonstrated in the spinal cords of sciatic nerve-injured animals, and the inhibition of these proteins also reduces mechanical and thermal hypersensitivity induced by nerve injury [6,18,19]. Nevertheless, the effects of sciatic nerve injury on the expression of PI3K/p-Akt and p-IκBα at the supraspinal level have not been completely assessed.

Oxidative stress is also involved in the development and maintenance of neuropathic pain. Nuclear factor erythroid derived-2-related factor 2 (Nrf2) and its downstream pathway, the enzymes heme oxygenase 1 (HO-1) and NAD(P)H:quinone oxidoreductase 1 (NQO1), are key to maintaining the homeostasis of organisms [20]. In vivo studies have demonstrated reduced Nrf2 and/or HO-1 levels in the spinal cord of animals with neuropathic pain [12,21] or diabetic neuropathy [22,23,24,25], in which the levels were restored by treatment with several pharmacological modulators of Nrf2 and HO-1 [12,26,27,28]. In addition, several compounds are capable of inhibiting inflammatory and neuropathic pain via inducing the Nrf2/HO-1 signaling pathway and inhibiting MAPK phosphorylation, cytokine production, and NF-κB activation in the spinal cord [8,22,23,24,25,26,27,28,29,30], but only a few studies have evaluated the antioxidant effects of these compounds in the hippocampus and prefrontal cortex of animals with chronic neuropathic pain. Similarly, recent studies have demonstrated that sulforaphane and cobalt protoporphyrin IX (CoPP), Nrf2 and HO-1 inducers, improve oxidative stress caused by nerve injury in the hippocampus and prefrontal cortex by activating the Nrf2 and HO-1 pathways [12,21,31]. A recent study further showed that another Nrf2 agent, dimethyl fumarate, inhibited neuropathic pain by activating hydroxyl carboxylic acid receptor type 2 [32].

Oltipraz, 5-(2-pyrazinyl)-4-methyl-1,2-dithiole-3-thione is an inducer of Nrf2 that has exhibited antioxidant, anticancer, and regenerative properties in different animal models and clinical trials by inducing the NQO1 isoenzyme [33,34,35,36]. Treatment with oltipraz also prevents insulin resistance and obesity associated with type 2 diabetes [37], and attenuates heart failure [38], renal and liver fibrosis [39,40], and kidney injury [41]. The effects of oltipraz on the nociceptive responses and depressive-like behaviors that accompany chronic pain and on microglial activation and subsequent induced pain signaling pathways in the spinal and supraspinal regions of sciatic nerve-injured animals have not yet been evaluated.

Here, we investigated the antinociceptive and antidepressant properties of oltipraz in a mouse model of neuropathic pain induced by the chronic constriction of the sciatic nerve (CCI). The effects of this drug on the expression of CD11b/c (a microglial marker), PI3K/p-Akt, p-IκBα (NF-κB), and MAPK, as well as that of Nrf2, HO-1, and NQO1, in the spinal cord, hippocampus, and prefrontal cortex of CCI-injured mice were evaluated 28 days after surgery.

## 2. Experimental Section

### 2.1. Animals

All experimental procedures were conducted with male C57BL/6J mice (21–25 g) obtained from Envigo Laboratories (Barcelona, Spain). The animals were housed under 12-h light/12-h dark conditions at 22 °C and 66% humidity and fed ad libitum. Before the experimental procedures, the mice were acclimated to these conditions for at least 7 days. The experiments were conducted between 9:00 a.m. and 5:00 p.m. All experiments were carried out in accordance with the guidelines of the European Commission’s directive (2010/63/EC) and Spanish Law (RD 53/2013) regulating animal research. In addition, all the experimental protocols were approved by the local Committee of Animal Use and Care of the Autonomous University of Barcelona (number: 1319). Maximal efforts were made to reduce animal suffering and the number of animals employed.

### 2.2. Induction of Neuropathic Pain

Neuropathic pain was induced with CCI according to our previous work [8]. Briefly, the animals were anesthetized with isoflurane (3% induction and 2% maintenance), and the right sciatic nerve was exposed by making an incision below the femur. The injury was created by tying three ligatures (4/0 silk, 1 mm between them) around the sciatic nerve, taking caution to preserve epineural circulation. The control mice (sham-operated) were exposed to the same surgical procedure without nerve ligation.

### 2.3. Nociceptive Testing

Mechanical allodynia was evaluated by measuring the hind paw withdrawal response to von Frey filaments. The mice were placed in Plexiglas boxes (20 cm high and 9 cm in diameter) with a wire grid bottom, through which the von Frey filaments (North Coast Medical, Inc., San Jose, CA, USA) were applied using the up-down paradigm [42]. A filament of 0.4 g was used first, and the strength of the next filament was increased or decreased depending on the response. The threshold of response was calculated using Excel (Microsoft Iberia SRL, Barcelona, Spain), and the calculation included the curve fitting of the data. Both the ipsilateral and contralateral hind paws were tested. The animals were habituated for 1 hour before testing to allow appropriate behavioral immobility.

Thermal hyperalgesia was evaluated by assessing the latency of paw withdrawal in response to radiant heat with the plantar test (Ugo Basile, Varese, Italy), according to [43]. The mice were placed in Plexiglas boxes (20 cm high and 9 cm in diameter) placed on a glass surface. The heat source was situated under the plantar surface of the hind paw and activated with a light beam intensity. To avoid tissue damage due to the lack of a response, a cutoff time of 12 s was established. The mean paw withdrawal latency of both the contralateral and ipsilateral paws was obtained from the average of three distinct trials with a 5-min break between trials. The mice were habituated to the test for 1 hour before the experiment.

Thermal allodynia was measured using a cold plate apparatus (Ugo Basile, Varese, Italy) according to the method described in [44]. Each animal was placed over a plate cooled to 4 °C ± 0.5 °C, and the number of elevations of each hind paw was recorded over 5 min.

### 2.4. Measurement of Depressive-Like Behavior

The evaluation of depressive-like behavior was performed using the tail suspension test (TST) in accordance with the procedures described in [45] and the forced swimming test (FST) according to the method described in [46].

For the TST, each mouse was suspended 35 cm above the floor with adhesive tape attached to the tip of its tail. The entire experiment was recorded with a digital camera, and immobility time was measured over a period of 6 min. The mice were considered immobile when they remained completely motionless. For the FST, each mouse was placed in transparent Plexiglas cylinder (25 × 10 cm) containing water to a depth of 10 cm at 24 °C ± 0.1 °C. Each animal was subjected to forced swimming during 6 min and the total duration of immobility was measured during the last 4 min, when mice show a sufficiently stable level of immobility.

All these experiments were performed by experimenters blinded to the treatments applied.

### 2.5. Western Blot Analysis

The animals were euthanized by cervical dislocation 28 days after surgery (sham or CCI). The ipsilateral lumbar spinal cord, hippocampus, and prefrontal cortex were extracted, immediately frozen in liquid nitrogen and maintained at −80 °C until use. The tissues were homogenized in ice-cold lysis buffer (50 mM Tris Base, 150 nM NaCl, 1% NP-40, 2 mM ethylenediaminetetraacetic acid (EDTA), 1 mM phenylmethylsulfonyl fluoride, 0.5 Triton X-100, 0.1% sodium dodecyl sulfate, 1 mM Na_3_VO_4_, 25 mM NaF, 0.5% protease inhibitor cocktail, and 1% phosphatase inhibitor cocktail). All reagents were purchased from Sigma-Aldrich (St. Louis, MO, USA), except NP-40, which was acquired from Calbiochem (Darmstadt, Germany). The homogenate was solubilized for 1 hour at 4 °C, sonicated for 10 s, and centrifuged at 4° C for 15 min at 700× *g*.

The supernatants (60 µg of total protein) were mixed with 4× laemmli loading buffer and loaded onto 4% stacking/10% separating sodium dodecyl sulfate (SDS) polyacrylamide gels. The proteins were electrophoretically transferred onto a polyvinylidene fluoride membrane for 120 min, blocked for 1 hour and 15 min with phosphate buffered saline (PBS) plus 5% nonfat dry milk or with Tris buffered saline with Tween 20 plus 5% nonfat dry milk or 5% bovine serum albumin (BSA) and then incubated with specific rabbit primary antibodies against Nrf2 (1:160; ab62352, Abcam, Cambridge, U.K.), HO-1 (1:150; ab137749, Abcam, Cambridge, U.K.), NQO1 (1:250, N5288, Sigma-Aldrich, St. Louis, MO, USA), phosphorylated c-Jun N-terminal kinase (p-JNK) (1:250; 9251, Cell Signaling Technology, Danvers, MA, USA), total JNK (1:250; 9252, Cell Signaling Technology, Danvers, MA, USA), phosphorylated extracellular signal regulated kinase 1/2 (p-ERK 1/2) (1:250; 9101, Cell Signaling Technology, Danvers, MA, USA), total ERK 1/2 (1:250; 9102, Cell Signaling Technology, Danvers, MA, USA), phospho P38 (1:200; 9211, Cell Signaling Technology, Danvers, MA, USA), total P38 (1:200; 9212, Cell Signaling Technology, Danvers, MA, USA), PI3K (1:200; ab232997, Abcam, Cambridge, U.K.), phospho Akt (1:200; 9271, Cell Signaling Technology, Danvers, MA, USA), total Akt (1:200; 9272, Cell Signaling Technology, Danvers, MA, USA), phospho IκBα (1:150; ab133462, Abcam, Cambridge, U.K.), total IκBα (1:200; 4812, Cell Signaling Technology, Danvers, MA, USA), CD11b/c (1:200, NB110-40766, Novus Biologic, Litton, CO, USA), or glyceraldehyde-3-phosphate dehydrogenase antibody (GAPDH) (1:5000; ABS16, Merck, Billerica, MA, USA) overnight at 4 °C. A horseradish peroxidase-conjugated anti-rabbit secondary antibody (GE Healthcare, Little Chalfont, UK) was used to detect the proteins, which were visualized with chemiluminescence reagents (ECL kit; GE Healthcare, Little Chalfont, UK) and by exposure onto hyperfilm (GE Healthcare, Little Chalfont, UK). Band intensity was quantified by densitometry.

### 2.6. Experimental Procedures

In the CCI-injured and sham-operated mice, the nociceptive baseline responses were determined 17 days after surgery by the von Frey filaments test and the plantar and cold plate tests. Then, the animals were intraperitoneally injected with oltipraz or vehicle at a dose of 10 mg/kg from days 18 to 28 after surgery (11 days of repeated treatment) (*n* = 6 animals per group). On days 1, 4, 8, and 11 of treatment, the nociceptive responses were evaluated. In the CCI-injured and sham-operated mice treated with 10 mg/kg of oltipraz or vehicle for 11 consecutive days, depressive-like behaviors were also measured by the TST and FST (*n* = 8 animals per group). Finally, on day 28 after surgery, all animals were euthanized by cervical dislocation, and specific tissues were extracted to evaluate protein levels by Western blot. In these experiments, the sham-operated mice treated with vehicle were used as controls (*n* = 4–5 samples per group).

### 2.7. Drugs

Oltipraz was obtained from Merck Chemicals and Life Science S.A.U. (Madrid, Spain). It was dissolved in dimethyl sulfoxide (1.5% in 0.9% saline solution) and administered intraperitoneally at a dose of 10 mg/kg in a final volume of 10 mL/kg 3–4 hours prior to behavioral testing, in accordance to our preceding pilot studies and other experiments with Nrf2 activators [28]. The drug was prepared daily before administration. For each group treated with oltipraz, the respective control group received the same volume of vehicle.

### 2.8. Statistical Analyses

All data are expressed as the mean ± standard error of the mean (SEM). Statistical analysis was carried out using the SPSS program (version 13 for Windows, IBM, Madrid, Spain). The effects of repetitive treatment with oltipraz on the mechanical allodynia, thermal hyperalgesia, and thermal allodynia induced by CCI were evaluated by using three-way repeated measures analysis of variance (ANOVA) with surgery, treatment, and time as the factors of variation followed by one-way ANOVA and the Student-Newman-Keuls (SNK) test. The effects of oltipraz on depressive-like behaviors were assessed using two-way ANOVA (with surgery and treatment as factors) followed by one-way ANOVA and the SNK test. Changes in the protein levels were analyzed using one-way ANOVA followed by the SNK test. A value of *p* < 0.05 was considered significant.

## 3. Results

### 3.1. Treatment with Oltipraz Produces Antinociceptive and Antidepressant Effects in CCI-Injured Mice

**Figure 1:**
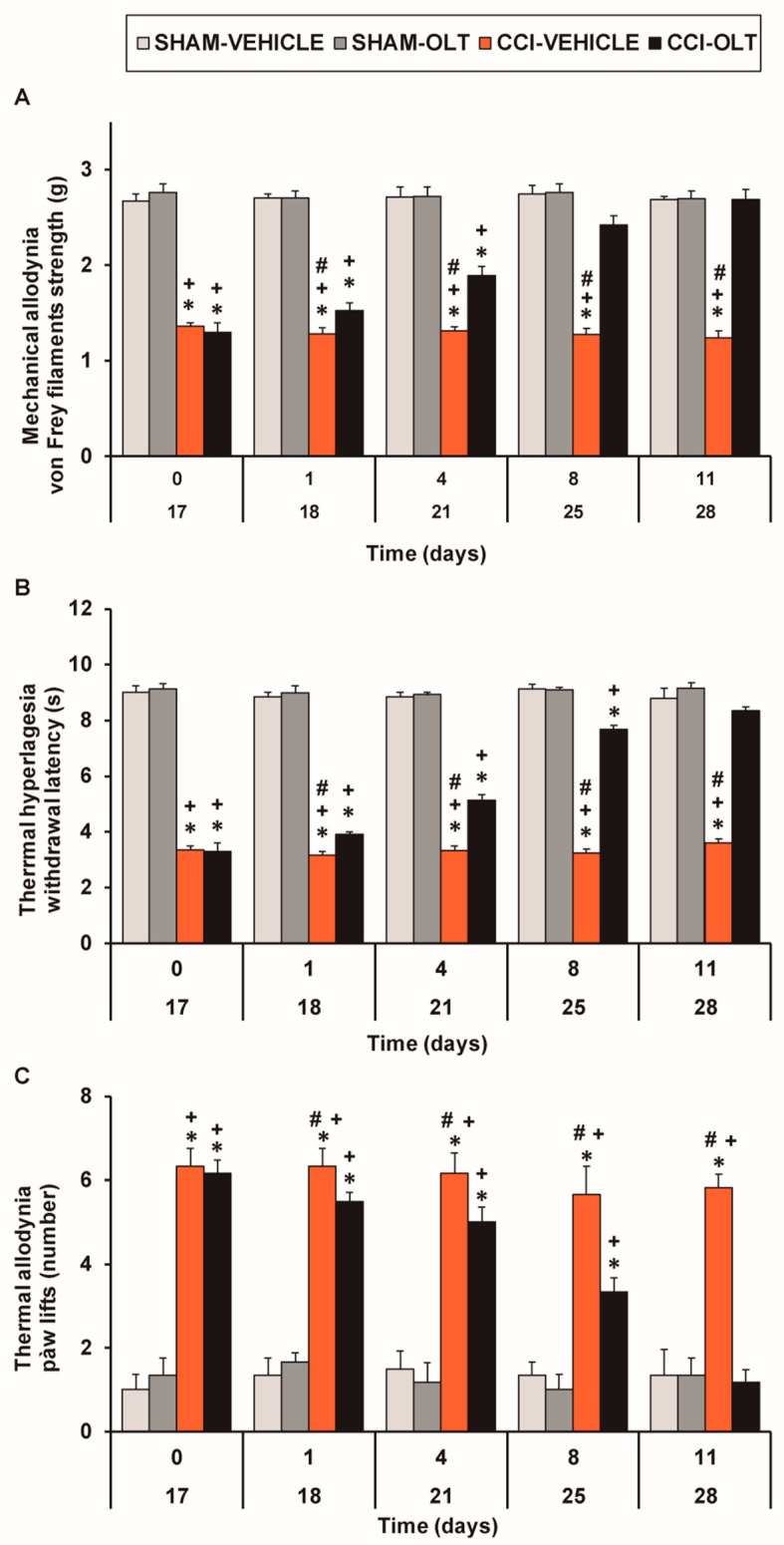
Repeated treatment with oltipraz reduces mechanical allodynia, thermal hyperalgesia, and thermal allodynia in CCI-injured mice. The development of (**A**) mechanical allodynia, (**B**) thermal hyperalgesia, and (**C**) thermal allodynia in the ipsilateral paw of the CCI-injured or sham-operated (SHAM) mice treated with 10 mg/kg oltipraz (OLT) or vehicle for 11 consecutive days is shown. The effects of oltipraz were evaluated at days 18, 21, 25, and 28 after surgery. For each test and time evaluated, * denotes significant differences vs. sham-operated mice treated with vehicle, + denotes significant differences vs. sham-operated mice treated with oltipraz, and # denotes significant differences vs. CCI-injured mice treated with oltipraz (*p* < 0.05; one-way ANOVA followed by the Student-Newman-Keuls (SNK) test). Results are presented as the mean ± standard error of the mean (SEM); *n* = 6–8 animals per experimental group.

For mechanical allodynia, three-way repeated measures ANOVA revealed significant effects of surgery (*F* (1,5) = 546.92, *p* < 0.001), treatment (*F* (1,5) = 142.32, *p* < 0.001), and time (F (4,20) = 22.93, *p* < 0.001) and interactions between surgery and treatment (*F* (1,5) = 160.65, *p* < 0.001), surgery and time (*F* (4,20) = 11.70, *p* < 0.001), treatment and time (*F* (4,20) = 21.29, *p* < 0.001), and between the three factors (*F* (4,20) = 21.52, *p* < 0.001). Our results confirmed that CCI reduced the threshold of ipsilateral hind paw withdrawal to von Frey filaments stimulation from days 17 to 28 after surgery (*p* < 0.001, one-way ANOVA followed by the SNK test vs. the corresponding sham-operated mice treated with vehicle; Figure 1A, Table 1). 

The repeated administration of oltipraz decreased the mechanical allodynia induced by CCI in a time-dependent manner, and after eight days of treatment, the threshold of ipsilateral paw withdrawal to a mechanical stimulus in the CCI-injured mice treated with oltipraz was similar to that obtained in the sham-operated animals treated with vehicle or oltipraz (Figure 1A).

In the plantar test, three-way repeated measures ANOVA also revealed significant effects of surgery (*F* (1,5) = 1630.62, *p* < 0.001), treatment (*F* (1,5) = 192.36, *p* < 0.001), and time (*F* (4,20) = 59.89, *p* < 0.001) and interactions between surgery and treatment (*F* (1,5) = 463.61, *p* < 0.001), surgery and time (*F* (4,20) = 61.25, *p* < 0.001), treatment and time (*F* (4,20) = 26.70, *p* < 0.001), and between the three factors (*F* (4,20) = 20.71, *p* < 0.001).

The CCI-induced decreased withdrawal threshold of the ipsilateral hind paw in response to a thermal stimulus from days 17 to 28 after surgery (*p* < 0.001, one-way ANOVA followed by the SNK test vs. the corresponding sham-operated mice treated with vehicle; Figure 1B and Table 1) was also inhibited by oltipraz treatment in a time-dependent manner. The thermal hyperalgesia induced by CCI was completely reversed after 11 days of treatment with oltipraz (Figure 1B).

Finally, regarding thermal allodynia, three-way repeated measures ANOVA demonstrated significant effects of surgery (*F* (1,5) = 421.49, *p* < 0.001), treatment (*F* (1,5) = 14.41, *p* < 0.013), and time (*F* (4,20) = 16.23, *p* < 0.001). A significant interaction between surgery and treatment (*F* (1,5) = 45.97, *p* < 0.001), surgery and time (*F* (4,20) = 11.18, *p* < 0.001), treatment and time (*F* (4,20) = 5.37, *p* < 0.004), and between the three factors (*F* (4,20) = 4.60, *p* < 0.008) was demonstrated. Consequently, the significantly increased number of ipsilateral hind paw lifts caused by cold stimulus in the CCI-injured mice from days 17 to 28 after surgery (*p* < 0.001, one-way ANOVA followed by SNK test vs. the corresponding sham-operated mice treated with vehicle; Figure 1C and Table 1) was reduced by oltipraz in a time-dependent manner and was completely normalized after 11 days of treatment. The intraperitoneal administration of oltipraz did not have any significant effects on nociception in either the ipsilateral paw of the sham-operated mice (Figure 1) or the contralateral paw of the CCI-injured or sham-operated animals.

In summary, these results indicate that the repeated intraperitoneal administration of oltipraz inhibited mechanical allodynia, thermal hyperalgesia, and thermal allodynia induced by CCI in the mice.

**Figure 2:**
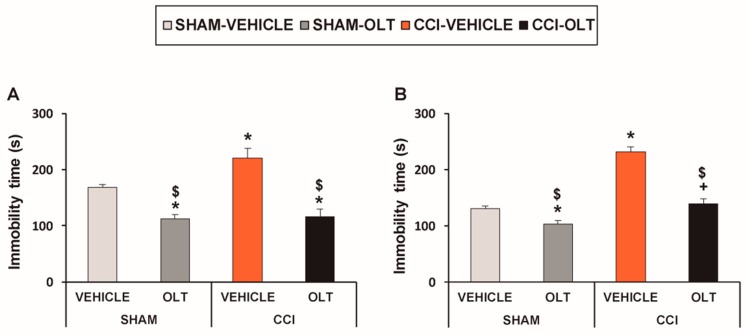
Repeated treatment with oltipraz reduces depressive-like behaviors in CCI-injured mice. Immobility time (s) evaluated by the (**A**) tail suspension test (TST) and (**B**) forced swimming test (FST) 28 days after surgery in the CCI-injured and sham-operated (SHAM) mice treated for 11 consecutive days with 10 mg/kg of oltipraz (OLT) or vehicle is shown. For each test evaluated, * denotes significant differences vs. sham-operated mice treated with vehicle, + denotes significant differences vs. sham-operated mice treated with oltipraz, and $ denotes significant differences vs. CCI-injured mice treated with vehicle (*p* < 0.05; one-way ANOVA followed by the SNK test). Results are presented as the mean ± SEM; *n* = 8 animals per experimental group.

Treatment with oltipraz also inhibited depressive-like behavior associated with neuropathic pain. For the TST, two-way ANOVA revealed significant effects of treatment (*F* (1,28) = 47.19, *p* < 0.001) and surgery (*F* (1,28) = 5.97, *p* < 0.021) and of the interaction between treatment and surgery (*F* (1,28) = 4.20, *p* < 0.05). The significant increase in immobility time observed in the CCI-injured animals treated with vehicle (*F* (3,28) = 19.12, *p* < 0.001, one-way ANOVA followed by the SNK test vs. the sham-operated mice treated with vehicle, Figure 2A) was significantly reduced by the repeated administration of oltipraz. This treatment also significantly reduced immobility time in the sham-operated mice (*F* (3,28) = 19.12, *p* < 0.001, one-way ANOVA followed by the SNK test vs. the sham-operated mice treated with vehicle), thus revealing the antidepressant effects of this drug in animals with and without chronic pain.

For the FST, two-way ANOVA also revealed significant effects of treatment (*F* (1,28) = 61.48, *p* < 0.001) and surgery (*F* (1,28) = 81.16, *p* < 0.001), and of the interaction between treatment and surgery (*F* (1,28) = 18.18, *p* < 0.001). In consequence, the significant increase in immobility time observed in the CCI-injured animals treated with vehicle (*F* (3,28) = 53.61, *p* < 0.001, one-way ANOVA followed by the SNK test vs. the sham-operated mice treated with vehicle, Figure 2B) was reduced by oltipraz. This treatment also significantly reduced immobility time in the sham-operated mice (*F* (3,28) = 53.61, *p* < 0.001, one-way ANOVA followed by the SNK test vs. the sham-operated mice treated with vehicle), thus confirming the antidepressant effects of this drug in animals with and without chronic pain.

### 3.2. Effect of Oltipraz on Expression of CD11b/c, PI3K/p-Akt, p-IkBα, Nrf2, HO-1, and NQO1 in Spinal Cord of CCI-Injured Mice

**Figure 3:**
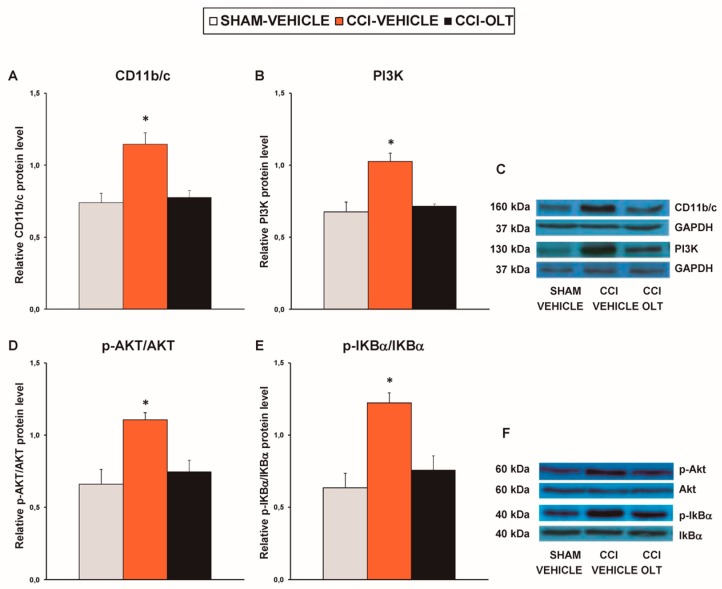
Effects of oltipraz on the expression of CD11b/c, phosphoinositide 3-kinase (PI3K), phosphorylated protein kinase B (p-Akt), and phosphorylated inhibitor of κBα (p-IkBα) in the spinal cord of the CCI-injured mice. The relative protein levels of (**A**) CD11b/c, (**B**) PI3K, (**D**) p-Akt, and (**E**) p-IKBα on the ipsilateral side of the spinal cord in the CCI-injured mice treated with oltipraz (OLT) or vehicle are represented. The sham-operated mice (SHAM) treated with vehicle were used as controls. (**C**) Representative examples of blots for CD11b/c (160 kDa), PI3K (130 kDa), and GAPDH (37 kDa), and (**F**) for p-Akt (60 kDa), Akt (60 kDa), p-IKBα (40 kDa) and IKBα (40 kDa). CD11b/c and PI3K are expressed relative to GAPDH levels whereas phosphorylated proteins are expressed relative to their corresponding total proteins. In all panels, * denotes significant differences vs. sham-operated mice treated with vehicle (*p* < 0.05; one-way ANOVA followed by the SNK test). Results are presented as the mean ± SEM; *n* = 5 samples per experimental group.

Our results revealed that sciatic nerve injury caused a significant increase in the expression of CD11b/c (*F* (2,12) = 11.82, *p* < 0.001, one-way ANOVA vs. the sham-operated mice treated with vehicle; Figure 3A), PI3K (*F* (2,12) = 14.93, *p* < 0.001, one-way ANOVA vs. the sham-operated mice treated with vehicle; Figure 3B), p-Akt (*F* (2,12) = 8.88, *p* < 0.004, one-way ANOVA vs. the sham-operated mice treated with vehicle; Figure 3D) and p-IKBα (*F* (2,12) = 14.88, *p* < 0.001, one-way ANOVA vs. the sham-operated mice treated with vehicle; Figure 3E) in the spinal cord. Treatment with oltipraz normalized the protein levels of CD11b/c, PI3K, p-Akt, and p-IKBα in this tissue.

**Figure 4:**
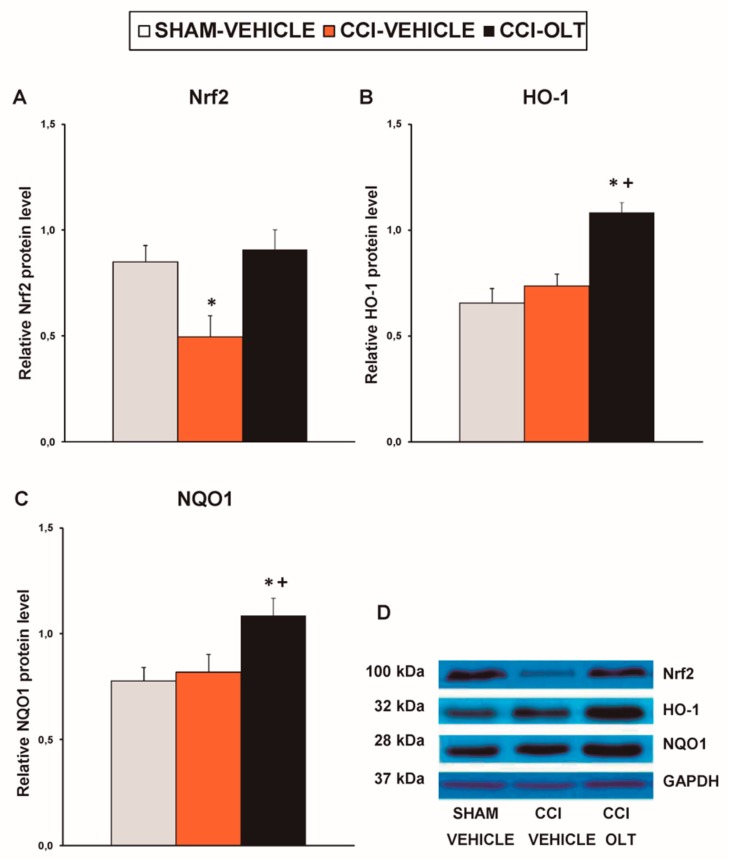
Effects of oltipraz on the expression of nuclear factor erythroid derived-2-related factor 2 (Nrf2), heme oxygenase 1 (HO-1) and NAD(P)H:quinone oxidoreductase 1 (NQO1) in the spinal cord of the CCI-injured mice. The relative protein levels of (**A**) Nrf2, (**B**) HO-1, and (**C**) NQO1 on the ipsilateral side of the spinal cord in the CCI-injured mice treated with oltipraz (OLT) or vehicle are represented. The sham-operated mice (SHAM) treated with vehicle were used as controls. (**D**) Representative examples of blots for Nrf2 (100 kDa), HO-1 (32 kDa), NQO1 (28 kDa) and GAPDH (37 kDa). Protein levels are expressed relative to GAPDH levels. In all panels, * denotes significant differences vs. sham-operated mice treated with vehicle and + denotes significant differences vs. CCI-injured vehicle-treated mice (*p* < 0.05; one-way ANOVA followed by the SNK test). Results are presented as the mean ± SEM; *n* = 5 samples per experimental group.

Sciatic nerve injury also decreased the expression of Nrf2 (*F* (2,12) = 7.59, *p* < 0.007, one-way ANOVA vs. the sham-operated mice treated with vehicle; Figure 4A) on the ipsilateral side of the spinal cord of the CCI-injured mice. No changes in the expression of HO-1 (Figure 4B) or NQO1 (Figure 4C) in this tissue were observed being induced by sciatic nerve injury. Treatment with oltipraz normalized the protein levels of Nrf2 and augmented the expression of HO-1 (*F* (2,12) = 15.88, *p* < 0.001, one-way ANOVA vs. the sham-operated and the CCI-injured mice treated with vehicle; Figure 4B) and NQO1 (*F* (2,12) = 4.79, *p* < 0.030, one-way ANOVA vs. the sham-operated mice treated with vehicle; Figure 4C) in the spinal cord of the CCI-injured mice.

### 3.3. Effect of Oltipraz on Expression of p-JNK, p-ERK ½, and p-P38 in Spinal Cord of CCI-Injured Mice

**Figure 5:**
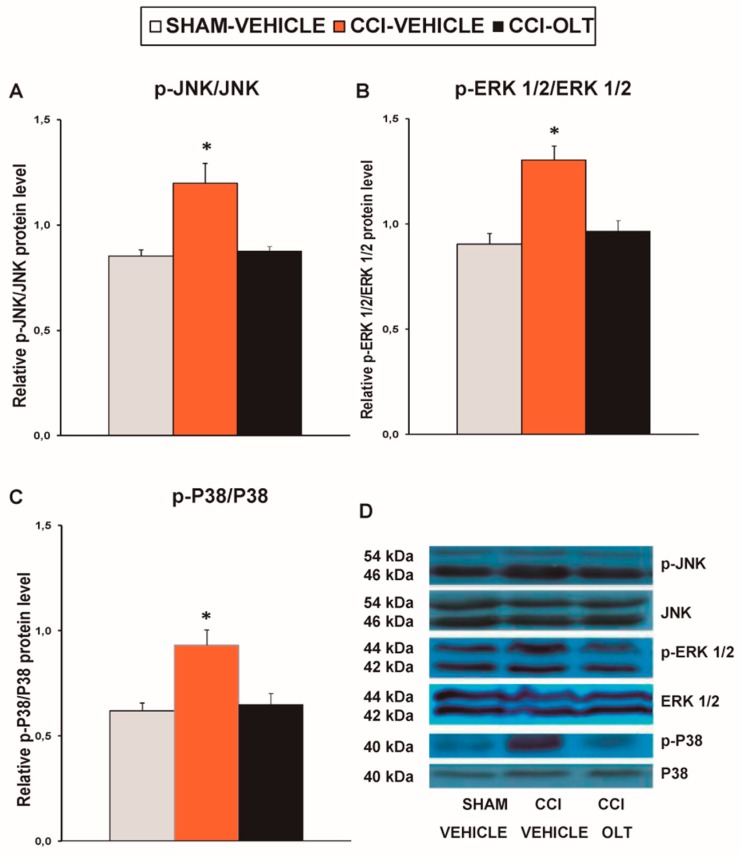
Effects of oltipraz on the expression of phosphorylated c-Jun N-terminal kinase (p-JNK), phosphorylated extracellular signal regulated kinase 1/2 (p-ERK 1/2), and p-P38 in the spinal cord of the CCI-injured mice. The relative protein levels of (**A**) p-JNK, (**B**) p-ERK 1/2, and (**C**) p-P38 on the ipsilateral side of the spinal cord of the CCI-injured mice treated with oltipraz (OLT) or vehicle are represented. The sham-operated mice (SHAM) treated with vehicle were used as controls. Phosphorylated proteins are expressed relative to their corresponding total protein levels. (**D**) Representative examples of blots for p-JNK/total JNK protein (46–54 kDa), p-ERK ½/total ERK ½ (42–44 kDa), and p-P38/total P38 (40 kDa). In all panels, * denotes significant differences vs. sham-operated mice treated with vehicle (*p* < 0.05; one-way ANOVA followed by the SNK test). Results are presented as the mean ± SEM; *n* = 5 samples per experimental group.

Sciatic nerve injury caused a significant increase in the expression of p-JNK (*F* (2,12) = 11.40, *p* < 0.002, one-way ANOVA vs. the sham-operated mice treated with vehicle; Figure 5A), p-ERK 1/2 (*F* (2,12) = 14.79, *p* < 0.001, one-way ANOVA vs. the sham-operated mice treated with vehicle; Figure 5B), and p-P38 (*F* (2,12) = 12.53, *p* < 0.001, one-way ANOVA vs. the sham-operated mice treated with vehicle; Figure 5C) in the spinal cord, and these changes were reversed by oltipraz treatment.

### 3.4. Effect of oltipraz on Expression of CD11b/c, PI3K/p-Akt, p-IkBα, Nrf2, HO-1, and NQO1 in Hippocampus of CCI-Injured Mice

**Figure 6:**
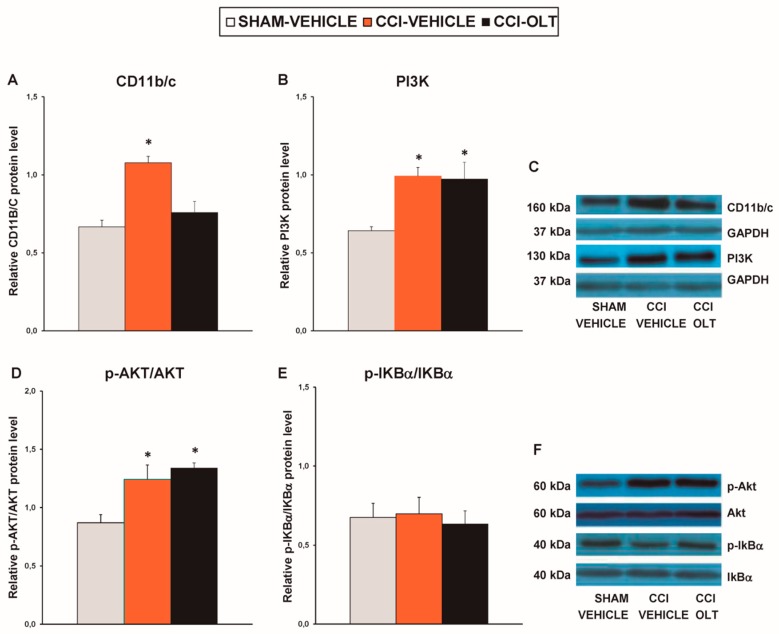
Effects of oltipraz on the expression of CD11b/c, PI3K, p-Akt, and p-IkBα in the hippocampus of the CCI-injured mice. The relative protein levels of (**A**) CD11b/c, (**B**) PI3K, (**D**) p-Akt, and (**E**) p-IKBα in the hippocampus of the CCI-injured mice treated with oltipraz (OLT) or vehicle. The sham-operated mice (SHAM) treated with vehicle were used as controls. Representative examples of blots for (**C**) CD11b/c (160 kDa), PI3K (130 kDa), and GAPDH (37 kDa); and (**F**) for p-Akt (60 kDa), Akt (60 kDa), p-IKBα (40 kDa), and IKBα (40 kDa). CD11b/c and PI3K are expressed relative to GAPDH levels whereas phosphorylated proteins are expressed relative to their corresponding total proteins. In all panels, * denotes significant differences vs. sham-operated mice treated with vehicle (*p* < 0.05; one-way ANOVA followed by the SNK test). Results are presented as the mean ± SEM; *n* = 5 samples per experimental group.

Our findings demonstrated that sciatic nerve injury increased the protein levels of CD11b/c (*F* (2,9) = 8.47, *p* < 0.009, one-way ANOVA vs. the sham-operated mice treated with vehicle; Figure 6A), PI3K (*F* (2,9) = 7.64, *p* < 0.011, one-way ANOVA vs. the sham-operated mice treated with vehicle; Figure 6B), and p-Akt (*F* (2,12) = 20.59, *p* < 0.001, one-way ANOVA vs. the sham-operated mice treated with vehicle; Figure 6D) in the hippocampus. Sciatic nerve injury did not change the expression of p-IKBα in the hippocampus (*F* (2,9) = 0.50, *p* > 0.05, one-way ANOVA; Figure 6E). Treatment with oltipraz inhibited the microglial activation but did not prevent the upregulation of PI3K or p-Akt in this brain area.

**Figure 7:**
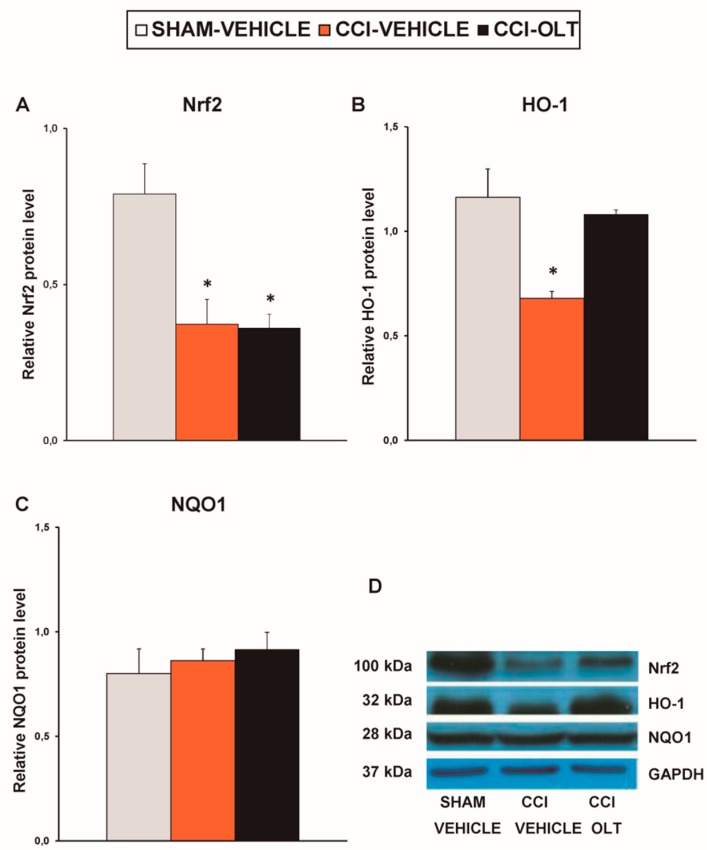
Effects of oltipraz on the expression of Nrf2, HO-1 and NQO1 in the hippocampus of the CCI-injured mice. The relative protein levels of (**A**) Nrf2, (**B**) HO-1, and (**C**) NQO1 in the hippocampus of the CCI-injured mice treated with oltipraz (OLT) or vehicle. The sham-operated mice (SHAM) treated with vehicle were used as controls. (**D**) Representative examples of blots for Nrf2 (100 kDa), HO-1 (32 kDa) and NQO1 (28 kDa) and GAPDH (37 kDa). Protein levels are expressed relative to GAPDH levels. In all panels, * denotes significant differences vs. sham-operated mice treated with vehicle (*p* < 0.05; one-way ANOVA followed by the SNK test). The results are presented as the mean ± SEM; *n* = 4–5 samples per experimental group.

Our findings also revealed that sciatic nerve injury decreased the expression of Nrf2 (*F* (2,9) = 6.30, *p* < 0.019, one-way ANOVA vs. the sham-operated mice treated with vehicle; Figure 7A) and HO-1 (*F* (2,9) = 10.07, *p* < 0.005, one-way ANOVA vs. the sham-operated mice treated with vehicle; Figure 7B). No changes in the expression of NQO1 (*F* (2,9) = 0.42, *p* > 0.05, one-way ANOVA; Figure 7C) were observed to be induced by CCI. Treatment with oltipraz normalized the down-regulation of HO-1 but not of Nrf2.

### 3.5. Effect of Oltipraz on Expression of CD11b/c, PI3K/p-Akt, p-IkBα, Nrf2, HO-,1 and NQO1 in Prefrontal Cortex of the CCI-Injured Mice

**Figure 8:**
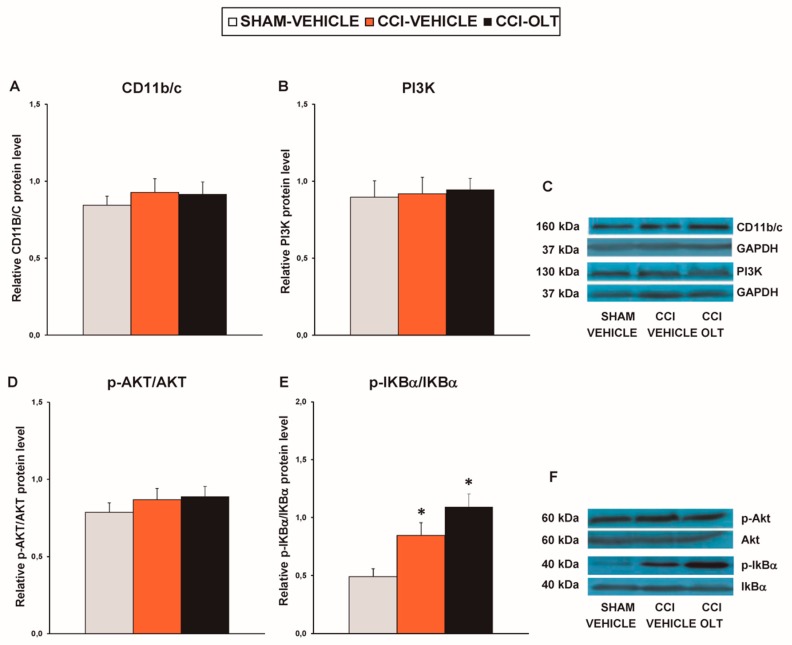
Effects of oltipraz on the expression of CD11b/c, PI3K, p-Akt, and p-IkBα in the prefrontal cortex of the CCI-injured mice. The relative protein levels of (**A**) CD11b/c, (**B**) PI3K, (**D**) p-Akt, and (**E**) p-IKBα in the prefrontal cortex of the CCI-injured mice treated with oltipraz (OLT) or vehicle. The sham-operated mice (SHAM) treated with vehicle were used as controls. Representative examples of blots for (**C**) CD11b/c (160 kDa), PI3K (130 kDa) and GAPDH (37 kDa), and for (**F**) p-Akt (60 kDa), Akt (60 kDa), p-IKBα (40 kDa) and IKBα (40 kDa). CD11b/c and PI3K are expressed relative to GAPDH levels whereas phosphorylated proteins are expressed relative to their corresponding total proteins. In all panels, * denotes significant differences vs. sham-operated mice treated with vehicle (*p* < 0.05; one-way ANOVA followed by the SNK test). Results are presented as the mean ± SEM; *n* = 4-–5 samples per experimental group.

The protein levels of CD11b/c (*F* (2,9) = 0.71, *p* > 0.05; Figure 8A), PI3K (*F* (2,9) = 1.12, *p* > 0.05; Figure 8B), and p-Akt (*F* (2,9) = 1.97, *p* > 0.05; Figure 8D) were not altered by sciatic nerve injury or by treatment with oltipraz. In contrast to what was found in the spinal cord, this treatment did not change the CCI-induced increased protein levels of p-IκBα (*F* (2,12)= 10.37, *p* < 0.002, one-way ANOVA vs. the sham-operated mice treated with vehicle; Figure 8E) in the prefrontal cortex.

**Figure 9:**
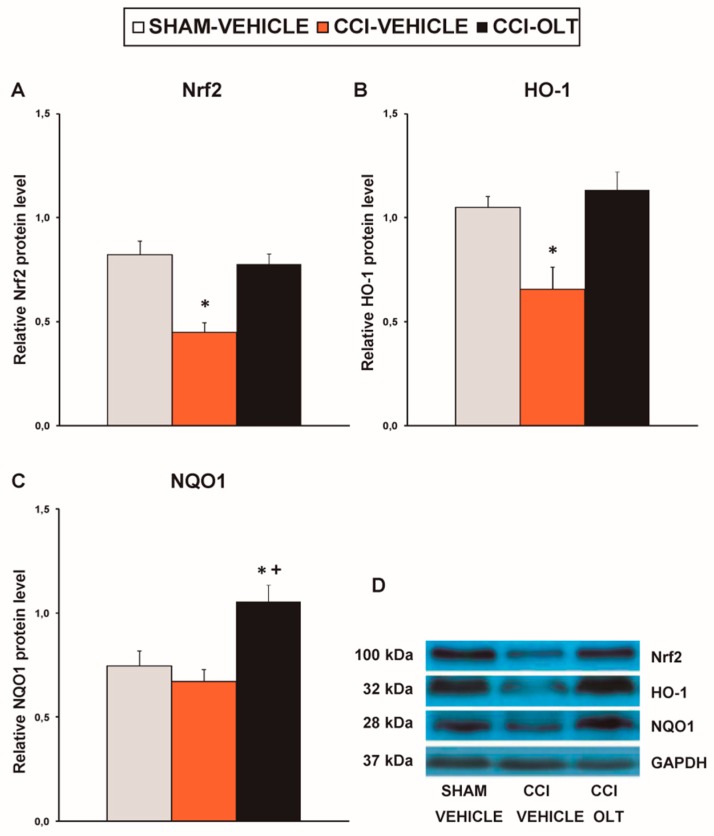
Effects of oltipraz on the expression of Nrf2, HO-1 and NQO1 in the in the prefrontal cortex of the CCI-injured mice. The relative protein levels of (**A**) Nrf2, (**B**) HO-1, and (**C**) NQO1 in the prefrontal cortex of the CCI-injured mice treated with oltipraz (OLT) or vehicle are represented. The sham-operated mice (SHAM) treated with vehicle were used as controls. (**D**) Representative examples of blots for Nrf2 (100 kDa), HO-1 (32 kDa) and NQO1 (28 kDa) and GAPDH (37 kDa). Protein levels are expressed relative to GAPDH levels. In all panels, * denotes significant differences vs. sham-operated mice treated with vehicle and + denotes significant differences vs. CCI-injured vehicle-treated mice (*p* < 0.05; one-way ANOVA followed by the SNK test). The results are presented as the mean ± SEM; *n* = 5 samples per experimental group.

Our data demonstrated a significant decrease in the expression of Nrf2 (*F* (2,12)= 14.41, *p* < 0.001, one-way ANOVA vs. the sham-operated mice treated with vehicle; Figure 9A) and HO-1 (*F* (2,12) = 9.20, *p* < 0.004, one-way ANOVA vs. the sham-operated mice treated with vehicle; Figure 9B) in the prefrontal cortex of the CCI-injured mice, and these changes were normalized by oltipraz. Although CCI did not alter NQO1 levels, a significant increase in the expression of this enzyme was revealed in the CCI-injured mice treated with oltipraz (*F* (2,12) = 8.45, *p* < 0.005, one-way ANOVA vs. the sham-operated and CCI-injured mice treated with vehicle; Figure 9C).

## 4. Discussion

This study demonstrated, for the first time, that treatment with oltipraz inhibited allodynia, hyperalgesia, and depressive-like behaviors induced by sciatic nerve injury by inhibiting microglial activation, avoiding the up-regulation of PI3K/p-Akt and p-IKBα, and activating the antioxidant enzymes HO-1 and NQO1 triggered by Nrf2 in the spinal cord, hippocampus, and/or prefrontal cortex.

The management of chronic neuropathic pain is a serious clinical problem due to the reduced efficacy of conventional treatments [47,48]. In this study, we demonstrated that the repetitive systemic injection of oltipraz for 11 days inhibited mechanical and thermal allodynia and thermal hyperalgesia caused by CCI in a time-dependent manner. These results are consistent with the antinociceptive effects of other Nrf2 inducers, including sulforaphane and plumbagin, and of several antioxidant compounds in animals with neuropathic pain induced by nerve injury, diabetes, or oxaliplatin [22,24,30,49,50,51,52], further demonstrating the antinociceptive properties of oltipraz under the condition of chronic neuropathic pain.

The participation of spinal microglial cells in the development of neuropathic pain via the activation of several intracellular paths, such as PI3K-, NF-κB-, and MAPK-related pathways, has been established. Consequently, the inhibition of microglial activation and/or the resulting activation of intracellular pathways is an effective approach to reducing neuropathic pain in its early stages of development, at 15 days post injury [3,4,6,18,19]. In this study, we also demonstrated that sciatic nerve injury activated microglia and neuroinflammation in the spinal cord 28 days after surgery. Augmented expression of CD11b/c (a microglial marker), PI3K/pAkt, p-IKBα, and MAPK (p-JNK, p-ERK1/2 and p-P38) was demonstrated in the spinal cord of the CCI-injured mice, supporting the contribution of these proteins to the maintenance of neuropathic pain [4,6,12,17]. Repetitive treatment with oltipraz inhibited microglial activation and neuroinflammation by blocking PI3K/Akt, IKBα, and MAPK upregulation in the spinal cord. These results reveal that the antinociceptive actions of oltipraz in chronic neuropathic pain may be produced via inhibiting microglial activation and neuroinflammation. Our results are in agreement with previous studies that observed the inhibition of microglia, MAPK, and cytokines induced by other Nrf2 transcription factor activators in the spinal cord of sciatic nerve-injured animals [30,31] and further demonstrate the participation of microglia, PI3K/p-Akt, p-IKBα, and MAPK in the analgesic effects induced by oltipraz in animals with persistent neuropathic pain.

The critical role of oxidative stress in the development of neuropathic pain has also been shown [12,51]. That is, Nrf2 coordinates the redox status of the organism through the upregulation of antioxidant genes, including HO-1 and NQO1 [53,54], but under neuronal injury, this homeostasis is disturbed, leading to exaggerated oxidative stress and neuropathic pain [12,22]. In agreement with these data, our findings show that CCI decreased the expression of Nrf2 in the spinal cord, confirming that nerve injury stimulates the prevailing oxidative stress conditions in this tissue [12]. Treatment with oltipraz reversed Nrf2 down-regulation and induced the overexpression of the antioxidant and detoxifying enzymes HO-1 and NQO1, suggesting that the analgesic action of oltipraz may also be mediated by activating the endogenous Nrf2/HO-1/NQO1 signaling pathway. In agreement with our findings, the prevention of insulin resistance and obesity induced by oltipraz in diabetic mice and the inhibition of neuropathic pain induced by sulforaphane in nerve-injured animals are also mediated by triggering the Nrf2/HO-1 pathway [31,37]. However, contrary to sulforaphane, oltipraz increases the expression of NQO1 in the spinal cord, suggesting that this detoxificant enzyme plays a critical role in the antinociceptive actions of oltipraz. Therefore, the antiallodynic and antihyperalgesic effects of oltipraz may be explained by its capacity to reestablish the homeostatic equilibrium via inhibiting activated microglia and subsequently triggering pain signaling pathways and activating antioxidant proteins.

Persistent neuropathic pain is associated with emotional disorders, such as depressive-like behaviors [55,56]. Our results confirmed this notion by showing that 28 days after sciatic nerve ligation, animals displayed depressive-like behavior manifested by an increase in the immobility time in TST and FST. The repeated administration of oltipraz inhibited this depressive-like behavior by reducing the immobility time in both tests. Our data also demonstrated the antidepressant effects of this drug in the sham-operated mice, which are in accordance with the antidepressant properties of other Nrf2 activators, such as TBE-31 and MCE-1, in different animal models of depression [57,58]. Microglial activation plays a crucial role in the pathogenesis of depression [59]. Activated microglia have been reported in different brain regions of depressive patients suffering chronic pain [60] and in animals with depressive-like behaviors associated with neuropathic pain [31]. Other studies have also demonstrated that the administration of microglial inhibitors, such as minocycline, relieves depressive-like behaviors in animals with chronic pain [61,62], supporting the contribution of microglia in the development of depressive-like behaviors. Our results reveal that sciatic nerve injury activated microglia and PI3K/p-Akt in the hippocampus and p-IKBα in the prefrontal cortex and thus contribute to central sensitization. The normalization of microglial activation induced by oltipraz in the hippocampus suggests that the antidepressant effects of this drug in the CCI-injured mice may have been produced via microglial inhibition. However, in contrast to what was found in the spinal cord, oltipraz did not inhibit PI3K/p-Akt in the hippocampus or p-IKBα in the prefrontal cortex; thus, the antinociceptive effects induced by oltipraz are mediated via the spinal cord regulation of these pain signaling pathways. Perhaps high doses of oltipraz or its central administration may be required to inhibit these central nociceptive pathways.

This study also demonstrated that CCI induces oxidative stress in the hippocampus and prefrontal cortex, as manifested by the diminished levels of Nrf2 and HO-1 in these brain areas. This concurred with the down-regulation of these enzymes observed in animals with depressive-like behaviors induced by chronic mild stress [63,64] or chronic pain [31]. Oltipraz normalized the down-regulation of Nrf2 in the prefrontal cortex and HO-1 in the hippocampus and prefrontal cortex, and also enhanced the expression of NQO1 in the prefrontal cortex. These results are in agreement with a study that showed the normalized expression of Nrf2 and HO-1 induced by sulforaphane, another Nrf2 activator, in these brain areas [31]; however, in contrast to sulforaphane, oltipraz also potentiated the overexpression of NQO1 in the prefrontal cortex, revealing the different mechanism of action of these compounds in neuropathic pain. Therefore, our findings showed, for the first time, that, in addition to Nrf2 and HO-1, NQO1 is also implicated in the central effects of oltipraz. These results are supported by the effects of other antidepressants, such as desipramine, which also inhibits depressive-like behavior provoked by chronic mild stress by activating both the HO-1 and NQO1 isoenzymes in the prefrontal cortex [64], and reveal the participation of NQO1 in the antidepressant effects of oltipraz.

In conclusion, this study showed that the intraperitoneal administration of oltipraz inhibited the mechanical and thermal sensitization induced by CCI by modulating the balance between oxidative stress and proinflammatory pathways triggered by microglia in the spinal cord. Our data also demonstrated the antidepressant properties of oltipraz in neuropathic pain, which may be mediated via inhibiting microglial activation in the hippocampus and triggering the Nrf2/HO-1/NQO1 signaling pathway in the prefrontal cortex and/or hippocampus. Thus, this study reveals that oltipraz may be an interesting target for neuropathic pain and depressive-like behavior management.

## Figures and Tables

**Table 1:** Summary of the one-way ANOVA performed with the data obtained for mechanical allodynia, thermal hyperalgesia, and thermal allodynia at different times (0, 1, 4, 8, and 11 days) after vehicle or oltipraz administration in the sham-operated and chronic constriction of the sciatic nerve (CCI)-injured mice.

Time of Treatment (Days)					
	0	1	4	8	11
**Mechanical**	F (3,20) = 96.8	F (3,20) = 124.9	F (3,20) = 124.2	F (3,20) = 60.9	F(3,20) = 180.6
**allodynia**	*p* < 0.001	*p* < 0.001	*p* < 0.001	*p* < 0.001	*p* < 0.001
**Thermal**	F (3,20) = 214.0	F (3,20) = 334.7	F (3,20) = 269.5	F (3,20) = 413.6	F(3,20) = 27.1
**hyperalgesia**	*p* < 0.001	*p* < 0.001	*p* < 0.001	*p* < 0.001	*p* < 0.001
**Thermal**	F (3,20) = 59.2	F (3,20) = 58.9	F (3,20) = 32.4	F (3,20) = 23.1	F(3,20) = 28.1
**allodynia**	*p* < 0.001	*p* < 0.001	*p* < 0.001	*p* < 0.001	*p* < 0.001
